# Effect of Core Training on Skill Performance Among Athletes: A Systematic Review

**DOI:** 10.3389/fphys.2022.915259

**Published:** 2022-06-06

**Authors:** Shengyao Luo, Kim Geok Soh, Kim Lam Soh, He Sun, Nasnoor Juzaily Mohd Nasiruddin, Congxin Du, Xiuwen Zhai

**Affiliations:** ^1^ Department of Sports Studies, Faculty of Educational Studies, Universiti Putra Malaysia, Serdang, Malaysia; ^2^ Department of Nursing, Faculty of Medicine and Health Sciences, Universiti Putra Malaysia, Serdang, Malaysia; ^3^ Department of Physical Education, Wuhan Sports University, Wuhan, China; ^4^ Department of Language and Humanities Education, Faculty of Educational Studies, Universiti Putra Malaysia, Serdang, Malaysia

**Keywords:** core training, skill performance, athletes, basketball, sport

## Abstract

**Background:** This study aims to present a critical review of the existing literature on the effect of core training on athletes’ skill performance, and to provide recommendations and suggest future research directions for both coaches and researchers.

**Methods:** The data in this study were reported using the Preferred Reporting Items for Systematic Reviews and Meta-Analyses (PRISMA) guideline. We collected studies in the literature using prominent academic and scientific databases such as Ebscohost, Scopus, PubMed, Web of Science, and Google Scholar. Only 16 of the 119 studies met all of the inclusion criteria, and were thus included in the systematic review. Each study’s quality was determined using the PEDro scale. The scoring of 16 studies ranges from 2 to 5.

**Results:** Core training could potentially improve skill performance among football, handball, basketball, swimming, dancing, Karate, Muay Thai, gymnasts, volleyball, badminton, and golf players.

**Conclusion:** Compared with the traditional training methods, core training is a new strength training method. Strong core muscles function as hubs in the biological motor chain, which create a fulcrum for the four limbs’ strength and establish a channel for the cohesion, transmission, and integration of the upper and lower limbs. In other words, core training optimizes the transfer and overall control of motion and force to the terminal segment within athletic actions. Meanwhile, core training could increase stability and stiffness in the spine to reduce unrequired “energy leaks” and torso movement during the exertion of external loads. This mechanism could help athletes achieve better skill performance. Therefore, this review suggests that core training should be considered integrated into athletes’ daily training routines.

**Systematic Review Registration**: [https://inplasy.com/], identifier [INPLASY2021100013].

## 1 Introduction

The most significant aspects impacting an athlete’s competitive abilities are his or her physical fitness and skills (Wang & Peng, 2007). However, prior research has mostly focused on anthropometric (e.g., length, wingspan, etc.) as well as physiological performance (e.g., agility, endurance, speed, and strength) (Koopmann et al., 2020). By comparison, the emphasis on these elements and the omission of other variables appears remarkable and results in significant unrealized potential. Skills appear to be particularly critical in light of the highly demanding and specialised proficiencies necessary for high performance in various sports (Wilson et al., 2017, 2019).

The sports field’s skill could be an event due to goals undertaken by a coordinated motor ability within a sport-specific scenario (Breivik, 2016). Scientific training is among the most critical factors that determine athletes’ overall performance (D'Isanto et al., 2019). Athletes constantly improve their skills through strength and conditioning programs (Viru & Viru, 2000; Reed et al., 2012).

In competitive sports, almost all sports exert force on external objects through the ends of human limbs to make the equipment move. Therefore, for a long time, coaches have mainly applied high-intensity strength training for limbs; the so-called traditional strength training (Li et al., 2008). Most traditional training approaches are not multi-planar and multi-articular, which splits the movement chain. These particular aspects are crucial for prompting improved performance (Fernandez-Fernandez et al., 2016; Fernandez-Fernandez et al., 2020). Another study showed that an unstable state of the body in the actual movement disallowed strength development in the stable state. As a result, it is difficult for the strength to influence movement during a play (Yu et al., 2008). Some athletes can show excellent strength during equipment strength training but cannot perform in the competition (Yu et al., 2008).

In the development of the movement chain theory, the human body can be divided into many links such as trunk, head, upper arm and forearm, thigh, calf, hand, and foot. Each link can be movably connected to form a movement chain (Hual et al., 2008). When the force acts on the movement chain, the relative position of each link will change, resulting in changes in human posture and movement state (Hual et al., 2008). Meanwhile, core muscles function as hubs in the biological motor chain, which create a fulcrum for the four limbs’ strength and establish a channel for the cohesion, transmission, and integration of the upper and lower limbs (Anant & Venugopal, 2021).

A simple analogy demonstrates the concept: even with the best tires, smoothest gearbox, and most streamlined body, a car is only as good as its motor, the powerhouse. Similarly, an athlete who lacks a stable and resilient core generates less than maximum output and falls well short of their athletic potential (Bliss & Teeple, 2005). As a result, core training has received increased attention as a means of enhancing athletes’ performance.

Since the early 1980s, core training, especially core stability training, has been the subject of many studies. Research has demonstrated the advantages of core training for patients suffering from back pain and daily activities. However, few studies concentrate on the impact of core training toward the performance of athletes (Ahmed et al., 2021). Until the 1990s, researchers investigated core muscle strength training for competitive sports (Li et al., 2008). So why could core training enhance athletes’ skill performance? Before this can be answered, core area, core strength, and core stability should be clarified. And what are the functions and differences between core stability and core strength when exercising?

The core can be thought of as an anatomical box composed of numerous muscle groups, including the abdominals in the front, the paraspinal and gluteal muscles in back, the diaphragm at the top, as well as the hip girdle and pelvic floor muscles at the bottom (Akuthota & Nadler, 2004; Shinkle et al., 2012). These muscles connect the lower and upper limbs by the spinal column and the thoracolumbar fascia. As a consequence, the core muscles and thoracolumbar fascia assist with rotation of the trunk and load transfer during exercise (Vleeming et al., 1995).

Core strength involves to the muscles’ capacity to generate force via contractile forces as well as intra-abdominal pressure (Faries & Greenwood, 2007). Core stability, on the other hand, refers to the ability of passive and active stabilisers in the lumbopelvic region to maintain reliable trunk and hip posture, stability, and control during static or dynamic movements. It is more vital than strength to a certain extent (Mendiguchia et al., 2011; Zazulak et al., 2007).

Competitive sports demand higher requirements for the body. Athletes need more complex and higher load core exercises to promote performance. Static low-intensity core stability training must be included in the core training, which looks more like the basis of the whole training plan (Faries & Greenwood, 2007). Core stability training could improve the ability of the nervous system to organize muscle coordination to enhance the efficiency of sports. Athletes will not be able to control and use the whole-body muscle strength well by ignoring core stability training, which may increase the risk of sports injury (Faries & Greenwood, 2007). Meanwhile, high load dynamic core strength training leads to muscle fiber hypertrophy and enhance muscle strength (Comerford, 2008). Therefore, based on above theories, a completed core training seems to include both two training models.

The media has mentioned the efficiency of “core training”, and that it could potentially improve their overall performance (Sharrock et al., 2011). However, the existing literature does not provide strong evidence for this view, especially in terms of skill performance. Therefore, the purpose of this study is to clarify the effect of core training on skill performance among athletes.

## 2 Meterials and Methods

### 2.1 Protocol and Registration

This review applied the Preferred Reporting Items for Systematic Reviews and Meta-Analyses (PRISMA) guideline to collect, select, and analyze the data, and was registered with the INPLASY website (https://inplasy.com/) (registration number: INPLASY2021100013; DOI number: 10.37766/inplasy 2021.10.0013) (Moher et al., 2010).

### 2.2 Search Strategy

Prominent academic databases were considered to search the related literature, including Ebscohost, Scopus, PubMed, Web of Science, as well as Google Scholar, until the end of 2021. For each independent database, a strategic search query was conducted by the title and abstract. The primary keywords considered for gathering related studies were: (“Core Strength Training” OR “Core-Muscle Training " OR “Core training” OR “Core-Stability Exercise” OR “Core Exercise”) AND (“Skill” OR “Skill Performance” OR “Sport Skill Performance” OR “Sport Technology")

### 2.3 Eligibility Criteria

The PICOS model was used for conducting the literature search. The acronym PICOS represents the following concepts: 1) population, 2) intervention, 3) comparison, 4) outcome, as well as 5) study design. The study employed each PICOS factor as an inclusion criterion for the publications that were searched. Each of the following inclusion requirements must be met for a study to be eligible:Table 1.

1) The study population must include healthy athletes, irrespective of gender or age.
2) Core training should be isolated and discussed explicitly, and the training during should be a minimum of 4 weeks.
3) The comparison in studies should be either single-group or multiple-group trials.
4) The study outcomes must comprise the impact of at least one core training exercise on the skill performance of athletes.
5) Articles must be experimental studies including Single-group Trials or Randomized Controlled Trials.

**TABLE 1:** PICOS Eligibility criteria.

PICOS	Detailed Information
Population	Healthy players
Intervention	Core training (not less than 4 weeks)
Comparison	Single-group trials, Two groups, and Three groups
Outcome	Include core training with various kinds of skill performance among players
Study Design	Single-group Trials or Randomized Controlled Trials

### 2.4 Study Selection

Two independent authors chose and included articles that met the stipulated inclusion criteria. To eliminate duplicates, this review employed the EndNote citation management system. The titles and abstracts of papers were assessed to determine which ones may be included in this research. In the event of a disagreement between the two authors over the selection of an article, a third author was consulted to analyse the entire piece and make a final decision.

### 2.5 Data Extraction and Quality Assessment

Post screening, some important data were obtained from qualified studies, namely, 1) Author name, year of publication; 2) Population characteristics (number, type, gender, age, gender); 3) Intervention features (type, measures index, frequency, and duration), and 4) Research outcomes.

The PEDro scale has good validity and reliability and has been proved to be a reliable metric for the methodology quality in constructing a systematic review (Lima et al., 2013). This scale contains 11 items with scores ranging from 0 to 10. Two independent raters use “yes” (1 point) or “no” (0 points) to evaluate these 11 items, respectively. If there are differences in the scoring process, the third rater will solve them. However, since it is related to external effectiveness, the score of eligibility criteria will not be included in calculating the total score. The higher the score, the better the quality of the methods.

## 3 Results

### 3.1 Study Selection

**FIGURE 1:**
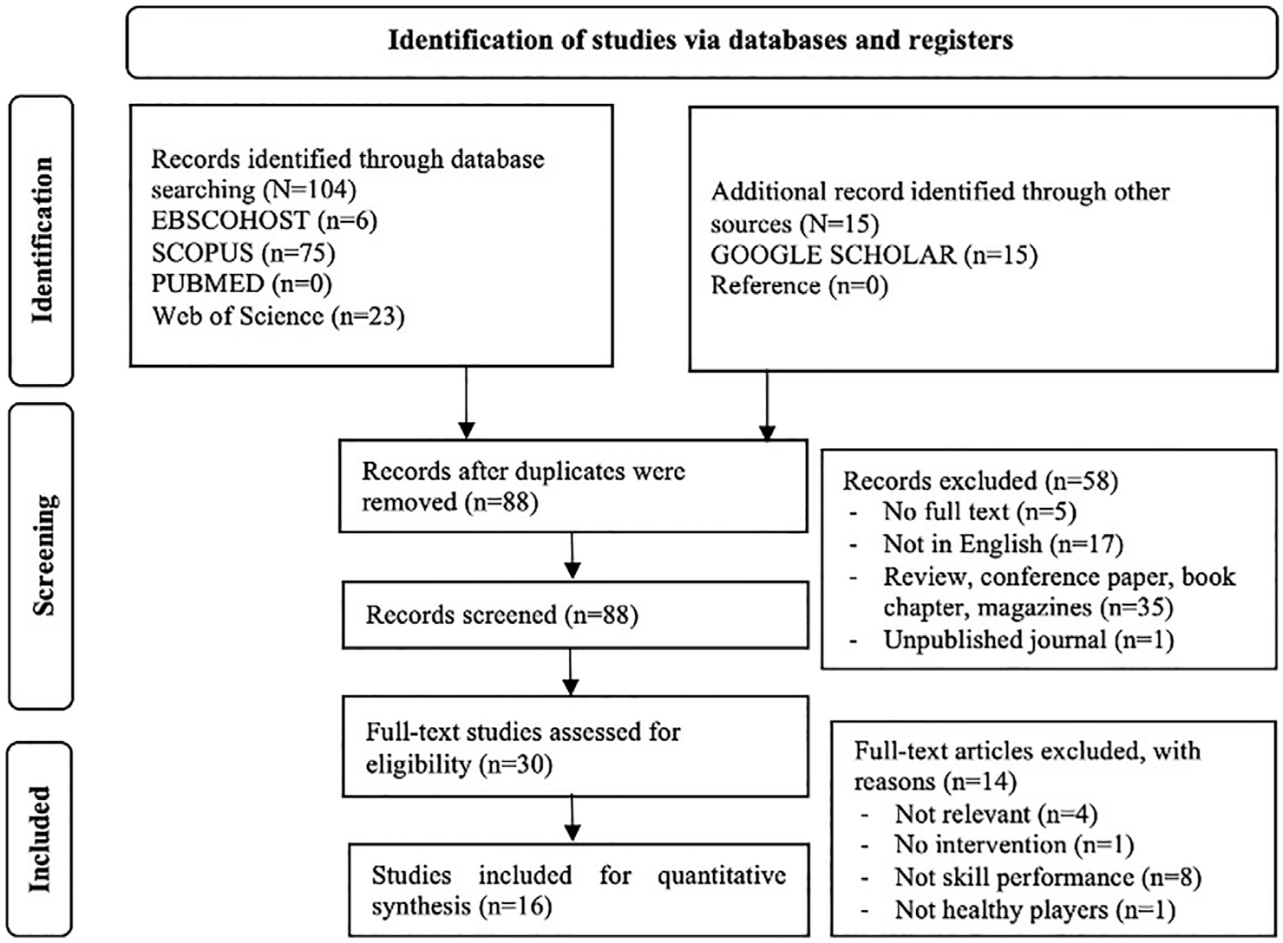
The search, screening and selection processes for suitable studies–based on PRISMA.

The technique of screening the literature is depicted in Figure 1. A total of 88 articles were retrieved after preliminary screening. This was obtained after Endnote software deleted duplicate articles, followed by a second round of deletions, which included five articles without full-text, 17 articles not in the English language, 35 articles not from journals, and one paper that was never published. Thirty full-text articles were evaluated for eligibility in the third screening phase. Four articles were omitted because they were unrelated to the topic area, and the final ten articles were also discarded. A total of 16 relevant publications satisfied the criteria for inclusion and were thus considered for the qualitative synthesis.

### 3.2 Study Quality Assessment

Table 2 provided detailed information for the PEDro scale score of each study. Data from all studies scored 2 to 5 on the PEDro scale. All studies had points deducted due to criteria relating to concealed allocation, blinding participants, assessors, therapists, and intention to treat analysis. Since the intervention is strength training, accompanied by professionalism and the risk of sports injury, it is difficult to blind participants, assessors, and therapists. However, studies could attempt to ensure all subjects received treatment.

**TABLE 2:** Summary of methodological quality assessment scores.

Study	Eligibility Criteria	Random Allocation	Allocation Concealment	Baseline Comparability	Blind Participants	Blind Therapist	Blind Assessor	Follow-Up	Intention to Treat Analysis	Between Group Comparisons	Point Measure and Variability	Total PEDro Score
Todd Watson et al. (2017)	1	0	0	0	0	0	0	1	0	0	1	2
Carmen Manchado et al. (2017)	1	1	0	1	0	0	0	1	0	1	1	5
Omaima Kamal (2015)	1	0	0	1	0	0	0	1	0	1	1	4
Benjamin Lee & Stuart McGill (2017)	1	0	0	0	0	0	0	1	0	1	1	3
Özgur Dogan and Seyfi Savas (2021)	0	0	0	1	0	0	0	1	0	1	1	4
Khaled Abuwarda (2014)	0	1	0	0	0	0	0	1	0	1	1	4
Mohamed Hamido Mahmoud (2018)	0	0	0	0	0	0	0	1	0	0	1	2
Shaheed Ahmed et al. (2021)	0	1	0	1	0	0	0	1	0	1	1	5
Özhan Bavlı & Caner Batuhan Koç (2018)	0	0	0	1	0	0	0	1	0	1	1	4
Ayşegül Yapıcı (2019)	0	0	0	1	0	0	0	1	0	1	1	4
Vesile Şahiner & Feyzullah Koca (2021)	0	0	0	1	0	0	0	1	0	1	1	4
Faruk Akçınar & Suat Macit (2020)	0	0	0	1	0	0	0	1	0	1	1	4
Ibrahim Hamed Ibrahim Hassan (2017)	1	1	0	1	0	0	0	1	0	1	1	5
Matthew Weston et al. (2013)	1	0	0	1	0	0	0	1	0	1	1	4
Ranya Mohamed Saeed Mahmoud (2021)	0	0	0	1	0	0	0	1	0	0	1	3
Dnyanesh Patil et al. (2012)	1	1	0	1	0	0	0	1	0	1	1	5

### 3.3 Participant Characteristics

Table 3 summarizes the participant’s characteristics for the sixteen studies which met the inclusion criteria, and these properties were highlighted as follows. 1) Categorization of athletes. Among the 16 documents, there were three articles on football players (Bavli & Koç, 2018; Ahmed et al., 2021; Mahmoud, 2021); three articles on handball players (Manchado et al., 2017; Akçinar & Macit, 2020; Mahmoud, 2021); two articles on basketball players (Dogan & Savaş, 2021; Şahiner & Koca, 2021); one article on swimmers (Patil et al., 2012); and the remaining seven articles were on dance (Watson et al., 2017), Karat (Kamal, 2015), Muay Thai (Lee & McGill, 2017), gymnasts (Abuwarda, 2014), volleyball (Yapıcı, 2019), badminton (Hassan, 2017) and golf (Weston et al., 2013). 2) Number, gender, and age. The total number of participants was 419, including 224 males and 167 females, and the remaining 28 participants did not report gender (Abuwarda, 2014; Mahmoud, 2018). Most studies reported the participants’ age, except for one study (Ahmed et al., 2021). Participants’ maximum and minimum age was 59 and 7 respectively, and the average age was 26.

**TABLE 3:** Population, Intervention and Main outcome.

Study	Population	Intervention						Main outcome related to skills
	N	Type of athletes	Gender	Age	Type	Skill measured index	Frequency & duration	
Todd Watson et al. (2017)	24	Dancers	Female	19.7 ± 1.1 years	EG: Core exercise	Pirouette ability	3 times/week 9 weeks	pirouette ability ↑
Carmen Manchado et al. (2017)	30	Handball players	Male	18.7 ± 3.4 years	EG: Core training CG: Routine training	Throwing velocity	4 times/week 10 weeks	Throwing velocity ↑
Omaima Kamal (2015)	20	Karate athletes	Female	12.54 years	EG: Core strength training CG: Traditional exercise	Spinning Wheel Kick	10 weeks	Spinning wheel kick ↑
Benjamin Lee & Stuart McGill (2017)	12	Muay Thai athletes	Male	24 ± 3 years	Group 1: Isometric core exercise Group 2: Dynamic core exercise Group 3: Routine training	Peak strike velocity and Peak impact force in Jab, Cross, Jab–Cross Combination (“Combo”), and Knee	6 weeks	Peak strike velocity and Peak impact force in Jab, Cross, Jab–Cross Combination (“Combo”), and Knee ↑
Özgur Dogan and Seyfi Savas (2021)	30	Basketball players	Male	12–14 years	EG: Core strength training CG: Routine training	Dribbling with right hand, Chest pass, V-Cut, taking pass, Jab-step, Dribbling and right layup, Rebound, Overhead pass, Cut towards left, Taking pass and dribbling with left hand, Dribbling with left hand between the legs, Cross over dribbling (right/left), Dribbling with right hand between the legs, Left lay-up, Cross over dribbling behind the back with left hand, Reverse dribbling with right hand, Hesitation (Dribbling), and Jump shot	8 weeks 3 times/week	Dribbling with right hand ↑ Chest pass ↑ V-Cut ↑ Taking pass ↔ Jab-step ↑ Dribbling and right layup ↔ Rebound ↑ Overhead pass ↑ Cut towards left ↑ Taking pass and dribbling with left hand ↑ Dribbling with left hand between legs ↑ Cross over dribbling (right/left) ↑ Dribbling with right hand between the legs↑ Left lay-up ↑ Cross over dribbling behind the back with left hand ↑ Reverse dribbling with right hand, Hesitation (Dribbling) ↑ Jump shot ↑
Khaled Abuwarda (2014)	12	Gymnasts	unknown	Under 7 years	EG: Core stability exercise CG: Routine training	Floor exercise, Vault Table	12 weeks 4 times/week	Floor exercise ↑ Vault Table ↑
Mohamed Hamido Mahmoud (2018)	16	Soccer players	unknown	15.40 ± 0.5 years	EG: Core training	Slalom dribble Lob pass, Shooting, Juggling	2 times/week 10 weeks	Slalom dribble ↑ Lob pass ↑ Shooting ↑ Juggling ↑
Shaheed Ahmed et al. (2021)	30	Football players	Male	unknown	EG: core training CG Conventional training	Juggling (foot) Juggling (body)	3 times/week 4 weeks	Juggling (foot) ↑ Juggling (body) ↑
Özhan Bavlı & Caner Batuhan Koç (2018)	18	Footballers	Male	15 ± 0.8 years	EG 1: dynamic core exercise EG 2: static core exercise CG: Routine training	Passing, Shooting, Dribbling	3 times/week 8 weeks	Passing ↑ Shooting ↑ Dribbling ↑
Ayşegül Yapıcı (2019)	32	Volleyball players	Female	16.62 ± 1.04 years	EG: core training CG Routine training	Service accuracy Velocity of the service	6 weeks	Service accuracy ↑ Velocity of the service ↑
Vesile Şahiner & Feyzullah Koca (2021)	22	Basketball players	Male	16–18 years	EG: core training CG Routine training	Free throw	2 times/week 8 weeks	Free throw ↑
Faruk Akçınar & Suat Macit (2020)	26	Handball athletes	Male	9–10 years	EG: Core training CG Routine training	Dribbling, Quick Pass, Point shooting	3 times/week 8 weeks	Dribbling ↑ Quick Pass ↑ Point Shooting ↑
Ibrahim Hamed Ibrahim Hassan (2017)	20	Badminton Players	Male	Under 19 years	EG: Core stability training CG Routine training	Smashing velocity, Smashing Accuracy	2 times/week 8 weeks	Smashing velocity ↑ Smashing Accuracy ↑
Matthew Weston et al. (2013)	36	Golf players	Male	47 ± 12 years	EG: Core training CG Routine training	Club-head speed, Backspin, and Sidespin	3 times/week 8 weeks	Club-head speed ↔ Backspin ↔ Sidespin ↔
Ranya Mohamed Saeed Mahmoud (2021)	7	Handball players	Female	12.3 ± 0.03 years	EG: Core strength training	Long jump shot, Fly distance, and long jump shot accuracy	3 times/week 10 weeks	Long jump shot ↑ Fly distance ↑ Long jump shot accuracy ↑
Dnyanesh Patil et al. (2012)	60	Swimmers	Mixed	12–18 years	EG: Core training CG Routine training	Stroke rate, Stroke length, Swim velocity, Stroke index	3 times/week 6 weeks	Stroke rate ↔ Stroke length ↔ Swim velocity ↑ Stroke index ↑ 50 m Freestyle ↑

EG, refers to Experimental group; CG, control group; CG1, control group one; ↑, significant improvement; ↔, no significant difference.

### 3.4 Intervention Characteristics

Table 3 shows several essential components of the study’s intervention characteristics, including the type of intervention, duration, and frequency. In terms of the intervention type, all works used core training as the primary intervention. Some studies refer to core training core exercise (Watson et al., 2017) as core strength training (Kamal, 2015; Dogan & Savaş, 2021; Mahmoud, 2021), isometric/dynamic core exercise (Lee & McGill, 2017), core stability exercise (Abuwarda, 2014), dynamic/static core exercise (Bavli & Koç, 2018), core stability training (Hassan, 2017). In addition, with the exception of three articles that only explored the effect of core training on athletes’ skill performance (Kamal, 2015; Lee & McGill, 2017; Yapıcı, 2019), the remaining studies compared the differences among core training and traditional strength training or routine training on participants’ skill performance.

As for duration, all studies lasted from four to 12 weeks. In these studies, most experiments lasted for 8 weeks (Weston et al., 2013; Hassan, 2017; Bavli & Koç, 2018; Akçinar & Macit, 2020; Dogan & Savaş, 2021; Şahiner & Koca, 2021), four studies lasted for 10 weeks (Kamal, 2015; Manchado et al., 2017; Mahmoud, 2018; Mahmoud, 2021), three studies lasted for 6 weeks, and the remaining studies lasted for 4 weeks (Ahmed et al., 2021), 9 weeks (Watson et al., 2017), and 12 weeks (Abuwarda, 2014), respectively.

As for frequency, only three studies did not report the training frequency (Kamal, 2015; Lee & McGill, 2017; Yapıcı, 2019). The frequency in other 13 studies was from 2 to 5 times a week (Patil et al., 2012; Weston et al., 2013; Abuwarda, 2014; Hassan, 2017; Manchado et al., 2017; Watson et al., 2017; Bavli & Koç, 2018; Mahmoud, 2018; Akçinar & Macit, 2020; Ahmed et al., 2021; Dogan & Savaş, 2021; Mahmoud, 2021; Şahiner & Koca, 2021).

### 3.5 Outcome

Table 4 shows the detailed classification of sports. Sports could mainly be divided into two categories: one is physical ability leading, and the other is skill leading. In the dominant physical category, it can be further divided into three subcategories 1) fast strength sports: jumping, throwing, and weightlifting; 2) speed sports: short distance running (100m, 200m, 400 m); short swim (50m, 100 m); short distance speed skating (500 m); short distance cycling (200m, 1000 m); 3) endurance sports: medium/super long-distance walking, running, skating, swimming, skiing, road cycling, and boating. Meanwhile, the skill leading category could also be divided into five subcategories: 1) accuracy sports: shooting, archery, crossbow, and golf; 2) aesthetic sports: gymnastics, rhythmic gymnastics, figure skating, figure swimming, freestyle skiing, dance, and martial arts routines; 3) net/court sports: table tennis, badminton, tennis, volleyball; 4) invasion sports: football, handball, basketball, and ice hockey; 5) combat sports: judo, boxing, martial arts, fencing, Muay Thai, and karate (Tian & Liu, 1984; Stefani, 1999; Livingston & Forbes, 2016; Zhou, 2017; Yin et al., 2018).

**TABLE 4:** The categories of sports.

General Category	Subcategories		Main Items
Physical ability leading	Fast strength sports		Jumping, throwing and weightlifting
	Speed sports		Short distance running (100m, 200m, 400 m); Short swim (50m, 100 m); Short distance speed skating (500 m); Short distance cycling (200m, 1000 m)
	Endurance sports		Medium/super long-distance walking, running, skating; Medium/super long-distance swimming, skiing; Medium/super long-distance road cycling and boating


Skill leading	Performance	Accuracy sports	Shooting, archery, crossbow, and golf
		Aesthetic sports	Gymnastics, rhythmic gymnastics, figure skating, figure swimming, freestyle skiing, dance, martial arts routines, etc
	Competition	Net/Court sports	Table tennis, badminton, tennis, volleyball
		Invasion sports	Football, handball, basketball, ice hockey, etc
		Combat sports	Judo, boxing, martial arts, fencing, Muay Thai, karate, etc

Therefore, this study systematically summarizes and analyzes the experimental results of the 16 studies based on the above categories.

#### 3.5.1 Effect of Core Training on Speed Sports

Among 60 young swimmers aged 12–18 years, only one study examined the effect of core training on performance in a speed sport (Patil et al., 2012). During the experiment, five indicators were evaluated: stroke rate, length, stroke index, swing velocity, as well as the 50 m freestyle swim. The result in this study showed a statistically significant difference in the most tests across two time period (pre-intervention and post-intervention) for the experiment group in the 50 m freestyle sprint time (36.74 ± 6.76 vs 35.71 ± 6.52, *p* < 0.05), swim velocity (1.40 ± 0.22 vs 1.44 ± 0.23, *p* < 0.05), and stroke index (2.0 ± 0.84 vs 2.14 ± 0.88, *p* < 0.05). Meanwhile, in the post test, there is significant difference in the 50 m freestyle sprint time between experiment group and control group (35.71 ± 6.52 vs 35.33 ± 4.43, *p* < 0.05).

#### 3.5.2 Effect of Core Training on Accuracy Sports

Among 36 male golfers aged 47 ± 12 years, only one study examined the effect of core training on skill performance in an accuracy sport (Weston et al., 2013). The results indicated that core training had a marginal effect on club-head speed (3.6%) when compared to the control group. Meanwhile, after performing core training, there was a modest reduction in the variability of various measurements of golfers’ repeated club-head speed as well as backspin (-8.2%), indicating a more stable golf swing.

#### 3.5.3 Effect of Core Training on Aesthetic Sports

Two studies have explored the effect of core training on skill performance in an aesthetic sport among 24 female adult dancers and 12 gymnasts aged under 7 years (Abuwarda, 2014; Watson et al., 2017). A study revealed that the number of pirouettes could significantly improve after doing core exercise (2.21 ± 0.63 vs 2.55 ± 0.81, *p* < 0.05) (Watson et al., 2017). Meanwhile, in another study, the skills of floor exercises and vault tables were measured among gymnasts. In comparison to the control group, the results showed that the experiment group added core stability training positively affected the skill performance of floor exercise and vault table (Abuwarda, 2014).

##### 3.5.4 Effect of Core Training on Net/Court Sports

One study investigated the effect of core training on skill performance in net/court sport among 32 female volleyball players aged 16.62 ± 1.04 years (Yapıcı, 2019). This study demonstrated a statistically significant difference in the service accuracy score assessment, as well as the velocity of service performance for the pre- and post-test of the experimental group (3.70 ± 0.48 vs 5.54 ± 0.52, *p* < 05; 31.23 ± 1.59 vs 37.38 ± 2.33, *p* < 0.05)). Meanwhile, a statistically significant difference was observed in the service accuracy score assessment, as well as the velocity of service, for the post-test for the both the control and experimental groups (3.80 ± 1.24 vs 5.54 ± 0.52, *p* < 0.05; 31.15 ± 1.56 vs 37.38 ± 2.33, *p* < 0.05).

On the other hand, badminton also belongs to the net/court sports category. A study examined the impact of core training on overall skill performance among 20 badminton players aged under 19 years (Hassan, 2017). The result demonstrated that an 8 week duration of core stability training could significantly improve smashing velocity (16.60 ± 2.07 vs 21.30 ± 2.58, *p* < 0.05) and accuracy skill performance (16.60 ± 2.07 vs 21.30 ± 2.58, *p* < 0.05) between pre and post test in the experimental group.

#### 3.5.5 Effect of Core Training on Invasion Sports

Handball, basketball, and football games belong to the invasion sports category. In the above 16 studies, most explored the effect of core training on skill performance around these sports (Manchado et al., 2017; Bavli & Koç, 2018; Mahmoud, 2018; Akçinar & Macit, 2020; Ahmed et al., 2021; Dogan & Savaş, 2021; Mahmoud, 2021; Şahiner & Koca, 2021). On the one hand, three studies examined the impact of core training on overall skill performance among 30 adult males (Manchado et al., 2017), 26 young males (Akçinar & Macit, 2020), and seven young females (Mahmoud, 2021) handball players respectively. The result of the first group of studies showed a statistically significant difference for the pre- and post-test in the all throwing tests for a standing throw with no run-up from the seven m line with no goalkeeper available (76.1 ± 10.9 vs 80.0 ± 10.8, *p* < 0.001) and with a goalkeeper (75.5 ± 10.7 vs 79.4 ± 9.6, *p* < 0.001); from the 9 m line with no goalkeeper (77.8 ± 10.2 vs 80.8 ± 10.3, *p* < 0.01) and with a goalkeeper (77.2 ± 11.1 vs 79.6 ± 10.1, *p* < 0.05); from the 9 m line with no goalkeeper (77.8 ± 10.2 vs 80.8 ± 10.3, *p* < 0.005) and with a goalkeeper (77.2 ± 11.1 vs 79.6 ± 10.1, *p* < 0.05); and a jump throw with a run-up from 9 m without a goalkeeper (80.4 ± 9.1 vs 83.6 ± 8.6, *p* < 0.001) and with a goalkeeper (79.9 ± 8.6 vs 83.8 ± 8.8, *p* < 0.005) (Manchado et al., 2017). Meanwhile, another study showed that the experimental group doing core training is significantly superior in skill parameters regarding dribbling, fast passing, and shooting performances compared to the control group (Akçinar & Macit, 2020). In addition, a similar positive impact of core training could be seen in another study. The result showed that after doing core training, the skills in terms of the long jump shot and fly distance and long jump shot accuracy were significantly improved (1.88 ± 0.27 vs 2.05 ± 0.29, *p* < 0.05; 47.97 ± 9.17 vs 51.06 ± 8.77, *p* < 0.05, respectively) (Mahmoud, 2021).

On the other hand, two studies were related to basketball skill performance, involving 52 young male basketball players (Dogan & Savaş, 2021; Şahiner & Koca, 2021). In one study, after experiencing core training, the significant difference could be observed in all basketball skill parameters in terms of dribbling with right hand (3.44 ± 0.964 vs 3,94 ± 0.854, *p* < 0.05), chest pass (2.94 ± 0.929 vs 3.69 ± 0.793, *p* < 0.05), v-cut (3.19 ± 1.109 vs 3.75 ± 0.856, *p* < 0.05), taking pass (3.13 ± 1.204 vs 3.75 ± 1.183, *p* < 0.05), jab-step (2.13 ± 0.885 vs 3.25 ± 1.065, *p* < 0.05), dribbling and right layup (2.75 ± 0.775 vs 3.63 ± 1.147, *p* < 0.05), rebound (2.56 ± 0.964 vs 3.50 ± 1.095, *p* < 0.05), overhead pass (2.81 ± 0.911 vs 3.38 ± 0.957, *p* < 0.05), cut towards left (3.38 ± 0.719 vs 3.88 ± 1.088, *p* < 0.05), taking pass and dribbling with left hand (3.00 ± 0.894 vs 3.69 ± 1.078, *p* < 0.05), dribbling with left hand between the legs (2.63 ± 0.957 vs 3.63 ± 1.204, *p* < 0.05), cross over dribbling (right) (2.81 ± 1.047 vs 3.75 ± 1.125, *p* < 0.05), cross over dribbling (left) (2.69 ± 1.078 vs 3.75 ± 1.183, *p* < 0.05), dribbling with right hand between the legs (2.56 ± 1.031 vs 3.69 ± 1.138, *p* < 0.05), left layup (2.69 ± 0.873 vs 3.56 ± 0.964, *p* < 0.05), rebound (2.63 ± 0.957 vs 3.63 ± 0.957, *p* < 0.05), cross over dribbling behind the back with left hand (2.88 ± 0.806 vs 3.44 ± 0.892, *p* < 0.05), reverse dribbling with right hand (2.44 ± 1.153 vs 3.56 ± 0.892, *p* < 0.05), hesitation (dribbling) (2.94 ± 1.124 vs 3.69 ± 0.946, *p* < 0.05), jump shot (2.63 ± 0.885 vs 3.56 ± 0.964, *p* < 0.05), respectively. Meanwhile, in comparing the post-test averages between two groups, except for skills of taking passes, dribbling and left lay-up, significant differences were found in all other skill parameters in terms of dribbling with right hand (3.94 ± 0.854 vs 2.64 ± 0.745, *p* < 0.05), chest pass (3.69 ± 0.793 vs 2.00 ± 0.877, *p* < 0.05), v-cut (3.75 ± 0.856 vs 2.57 ± 0.756, *p* < 0.05), jab-step (3.25 ± 1.065 vs 2.43 ± 0.756, *p* < 0.05), rebound (3.50 ± 1.095 vs 2.07 ± 0.730, *p* < 0.05), overhead pass (3.38 ± 0.957 vs 2.21 ± 0.802, *p* < 0.05), cut towards left (3.88 ± 1.088 vs 2.71 ± 0.469, *p* < 0.05), taking pass and dribbling with left hand (3.69 ± 1.078 vs 2.57 ± 0.852, *p* < 0.05), dribbling with left hand between the legs (3.63 ± 1.204 vs 1.93 ± 0.730, *p* < 0.05), cross over dribbling (right) (3.75 ± 1.125 vs 1.86 ± 0.770, *p* < 0.05), cross over dribbling (left) (3.75 ± 1.183 vs 2.00 ± 0.784, *p* < 0.05), dribbling with right hand between the legs (3.69 ± 1.138 vs 2.14 ± 0.864, *p* < 0.05), left layup (3.56 ± 0.964 vs 1.79 ± 0.699, *p* < 0.05), rebound (3.63 ± 0.957 vs 1.71 ± 0.726, *p* < 0.05), cross over dribbling behind the back with left hand (3.44 ± 0.892 vs 2.43 ± 0.646, *p* < 0.05), reverse dribbling with right hand (3.56 ± 0.892 vs 1.57 ± 0.756, *p* < 0.05), hesitation (dribbling) (3.69 ± 0.946 vs 1.29 ± 0.611, *p* < 0.05), jump shot (3.56 ± 0.964 vs 2.14 ± 0.535, *p* < 0.05) (Dogan & Savaş, 2021). In addition, other studies showed that both core training and routine training methods could improve the free-throw skill between two groups. Data analysis found a significant difference for the experimental and control group for the free throw between pre-test and post-test (5.81 ± 0.968 vs 6.81 ± 0.750, *p* < 0.05; 1.71 ± 0.726 vs 1.79 ± 0.699, *p* < 0.05, respectively). However, there was no difference between both groups (Şahiner & Koca, 2021).

In addition, three studies examined the impact of core training on overall skill performance among 16 young, 30 males, and 18 young males football players, respectively (Bavli & Koç, 2018; Mahmoud, 2018; Ahmed et al., 2021). Four football-specific skills were tested in a study, including slalom dribble, lob pass, shooting, and juggling. The results demonstrated that was a significant difference between the pre- and post-test results for Slalom dribble (17.40 ± 1.10 vs 15.50 ± 1.30, *p* < 0.01); Lob pass (21 ± 4.60 vs 25.10 ± 3.40, *p* < 0.01); Shooting accuracy (21.70 ± 3.40 vs 25.80 ± 2.70, *p* < 0.01); Juggling (97.40 ± 70.40 vs 117.70 ± 53.30, *p* < 0.01) (Mahmoud, 2018). Meanwhile, in the second study, juggling (foot) and juggling (body) as skill performances were measured (Ahmed et al., 2021). A statistically significant difference was found in juggling (foot) and juggling (body) in the experimental groups between pre-test and post-test (9.60 ± 5.30 vs15.80 ± 4.26, *p* < 0.05; 8.73 ± 3.173 vs 13.67 ± 2.69, *p* < 0.05). Meanwhile, the similar result also could be found in the control group (11.53 ± 5.15 vs 13.80 ± 3.82, *p* < 0.05; 9.27 ± 2.89 vs 11.07 ± 2.98, *p* < 0.05). At the same time, there is no significant difference in the post-test between both groups (*p* > 0.05). In addition, a study divided participants into three groups which applied dynamic, static core exercise, and routine training, respectively (Bavli & Koç, 2018). There are three football skills in dribbling, shooting, and passing involved in measurement. After applying 8 weeks of intervention, the result showed that only the control group applying routine training could not show a significant difference in the shooting, dribbling, and pass tests between the pre- and post-test results (134 ± 6.1 vs 136 ± 6.5, *p* > 0.05; 21.8 ± 0.8 vs 21.7 ± 0.8, *p* > 0.05; 7 ± 0.1 vs 7.8 ± 1.6, *p* > 0.05, respectively). In contrast, there was a substantial difference in dribbling ability between pre- and post-test among participants who received dynamic core training (21.4 ± 0.5 vs 20.9 ± 0.4, *p* < 0.05). Additionally, it may have a similar beneficial effect on the dribbling (21.3 ± 0.8 vs 20.5 ± 0.8, *p* < 0.05) and passing ability (8.5 ± 1.5 vs 10.6 ± 1.1, *p* < 0.05) among players who receive static core training. When variance in three groups was compared between pre- and post-tests, there were no significant changes between static and dynamic strength training players, although both static and dynamic training groups performed significantly better than the control group on dribbling (20.9 ± 0.4 vs 21.7 ± 0.8, *p* < 0.05; 20.9 ± 0.4 vs 21.7 ± 0.8, *p* < 0.05) and passing (10.6 ± 1.1 vs 7.8 ± 1.6, *p* < 0.05; 10.5 ± 1.1 vs 7.8 ± 1.6, *p* < 0.05).

#### 3.5.6 Effect of Core Training on Combat Sports

A study examined the effect of core training on skill performance in combat sports among 20 young female karate athletes (Kamal, 2015). The results revealed that core training could positively affect skill performance among karate athletes. A significant difference was observed in the post test between both groups in the spinning wheel kick assessment (6.50 ± 0.05 vs 5.59 ± 0.06, *p* < 0.05).

In contrast, another study investigated the impact of core training on 12 adult male Muay Thai athletes. This study divides these athletes into three groups and applied different interventions in terms of isometric core exercise, dynamic core exercise, and routine training respectively (Lee & McGill, 2017). Four actions strikes, namely, Jab, Cross, Jab-Cross Combination (“Combo”), and Knee were measured by impact force, strike velocity, and electromyography in this study. The results found that isometric and dynamic core training increased impact force in almost all tests. After isometric core training, a significant difference could be observed in the Jab impact force (2539.3 ± 89.1 vs 3093.7 ± 69.4 N, *p* < 0.001), Knee impact force (8242 ± 132.3 vs 9482 ± 152.8 N, *p* < 0.05), and Cross impact force increased by 26%. A similar positive effect also found after dynamic core training in Jab impact force (2614.7 ± 493.1 vs 3199.6 ± 437.9 N, *p* < 0.05) and Knee impact force (7.1% increase). However, the control group applying routine training did not find significant impact force changes.

As for strike velocity, after the intervention, both isometric and dynamic training could increase the maximum limb velocity for nearly all strike assessments. After isometric ore training, the significant difference could be observed in the peak strike velocity of Cross trials (6.7 ± 0.7 vs 8.8 ± 0.9 m s^−1^, *p* < 0.01) and the second strike of Combo trials (7.1 ± 0.5 vs 9.0 ± 1.0 m s^−1^, *p* < 0.03). Meanwhile, in the dynamic group, the significant difference between pre and post-test could be observed in peak strike velocity of Jab trials, Cross trials, Combo trials, and Knee trials (4.5 ± 0.4.0 vs 5.8 ± 0.6.0 m s^−1^, *p* < 0.05; 6.6 ± 0.9 vs 12.1 ± 1.3 m s^−1^, *p* < 0.001; 7.6 ± 0.8 vs 10.4 ± 1.0 m s^−1^, *p* < 0.05; 7.8 ± 0.6–11.0 ± 1.0 m s^−1^, *p* < 0.05, respectively). However, no changes were observed for the control group when applying routine training.

In comparing the three groups, dynamic core training had the most significant impact on increasing strike velocity and the effect of isometric core training was better than the control group for all tests. To be specific, After dynamic core training, the significant change shown in the strike velocity of Cross trials (5.5 ± 0.6 m s^−1^ increase), first and second Comb trails (0.7 ± 0.08 and 2.8 ± 0.2 m s^−1^ increase), Jab (1.3 ± 0.1 m s −1 increase), and knee (3.2 ± 0.3 m s^−1^ increase). Meanwhile, the isometric core training group was better than the control group for Jab velocity (0.0 ± 0.04 m s^−1^ increase vs 0.5 ± 0.05 m s^−1^ increase), Cross trials (2.1 ± 0.3 m s^−1^ increase vs 0.3 ± 0.05 m s^−1^ increase), first and second Combo velocity (increased by 0.3 ± 0.05 and 1.9 ± 0.2 m s^−1^ vs decreased by 0.6 ± 0.1 and 0.5 ± 0.05 m s^−1^), and Knee velocity (1.2 ± 0.2 m s^−1^ increase vs 0.4 ± 0.1 m s^−1^ increase).

A more scientific result was provided by electromyography. For all trials, major increases in peak EMG amplitude levels were estimated for both isometric core as well as dynamic core training groups. During jab trials, cross trials, combo trials, and knee trials, the isometric core training group increased the EMG amplitude across all musculature by 37%, 34%, 17%, and 25%, respectively. In contrast, the dynamic core training group increased the EMG amplitude of all musculature by 35%, 35%, 23%, and 20% respectively. In addition, in comparing the three groups, both training types improved peak EMG amplitude levels more than the control group for nearly all muscles in all assessments. To be specific, both the isometric core training and dynamic core training group increased the overall peak EMG amplitude by 103% on average more than the control group. However, the result is hard to identify when comparing isometric and dynamic core training because some muscles were more stimulated from isometric core training, while others responded better to dynamic core training.

## 4 Discussion

### 4.1 Effect of Core Training on Speed Sports

Only one study empirically showed that core training could significantly improve swimmers’ swing velocity, stroke index, and 50 m freestyle (Patil et al., 2012). Some studies have shown that stroke speed may be affected by stroke length and rate (Craig & Pendergast, 1979; Craig et al., 1985). However, another study shows that “turning time” is a more significant factor affecting stroke speed (Patil et al., 2012).

A reasonable explanation for this may be that enhancing muscle strength in the core area enables athletes to maintain the required streamlined posture during swimming continuously. When swimmers kick and stroke, the vital core can make the transmission of strength more coordinated and economical to effectively produce powerful movement and shorten the swimming time (Patil et al., 2012). Meanwhile, due to the enhancement of core muscles, the athlete’s pelvis becomes more stable, which helps the athlete to turn their body faster and coordinate, and saves the turning time (Chek, 2005). In addition, the stroke index is a reliable indicator of swimming efficiency, and it could be computed by the average velocity times the stroke length. Therefore, when stroke velocity increases and the stroke length is steady, the stroke index increases (Costill et al., 1985).

On the other hand, functional training is similar to core training. D'Elia (2013) summarized the importance of functional training in a short sentence: “your strength only depends on your weakest link.” This opinion is almost the same as Solomonow et al. (1998) on the importance of core strength - a car is only as good as its motor, the powerhouse (D'Elia L, 2013; Solomonow et al., 1998). Thompson (2016) believed that functional training is a kind of strength training. Besides developing strength, balance, motor coordination, and endurance, also increases the ability of individuals to execute activities of daily living or more complex athletic actions (Thompson, 2016). Some scholars believed that developing core stability is a marked feature in the functional training program, because the stability of the core area can help maintain the correct posture pattern and ensure efficiency and safety when performing static or dynamic actions (McGill, 2010; Tomljanović et al., 2011; Heinrich et al., 2012). A study further systematically summarizes functional training and holds that the characteristics of functional training are integrated, multi-joint/multi-segment, asymmetrical, multi planes, acidic, intermittent, speed, and unstable movements that emphasize core stability (La Scala Teixeira et al., 2017). Therefore, based on above opinions, this study believed that compared with core training, functional training seems to be a broader concept and researchers could consider core training as a key part of functional training.

### 4.2 Effect of Core Training on Accuracy Sports

A study showed that core training could slightly reduce the variable nature of certain measures of golfers’ repetitive club-head speed as well as the backspin, which indicates a more stable swing (Weston et al., 2013). During the golf swing, athlete need to create the axial rotation of the upper trunk in relation to the pelvis, and, ideally, the shoulder and hip complex can rotate over 45° (Gluck et al., 2008). The study’s core activities were predominantly isometric, with the spine in a neutral alignment. As a result, the workouts are predicted to exert less stress on the spine and distribute it more uniformly than swing-specific activities. According to skill acquisition theory, the extent to which these training effects are transferred is expected to be significantly smaller (Lederman, 2010). This may reflect why it had a negligible effect in this study.

### 4.3 Effect of Core Training on Aesthetic Sports

A study revealed that core training could improve the ability of pirouettes (Watson et al., 2017). Some studies examined the function of certain abdominal muscles including the transverse abdominis, rectus abdominis, external oblique, internal oblique, erector spinae, quadratus lumborum, and latissimus dorsi could produce rotation movements and control external forces that cause rotation to the spine. Dancers performing diverse, complex technical skills such as pirouette revolutions require increased motor control, especially of the extremities, and stability of the spine by the core muscles group. This is because when a dancer pirouetting, the core muscles need to work both concentrically and eccentrically (Lott & Laws, 2012; Rickman et al., 2012).

In the entire core muscles group, the local stabilizing subsystem comprises the transversus abdominis, multifidus, internal oblique muscles located deep to the global system and provided for a dynamic segmental spinal stability (Huxel Bliven & Anderson, 2013). A study examined specifically targeting the transversus abdominis inferred to improve static and dynamic balance (Zazulak et al., 2007). Additionally, another study demonstrated that the transversus abdominis has a complex function in trunk rotation (Urquhart & Hodges, 2005). Therefore, more muscular core strength could translate to a more successful pirouette.

On the other hand, gymnasts need to complete all kinds of difficult somersaults and rotations in the air (Yang & Li, 2010). A study revealed that floor exercises and vault table skills were improved among gymnasts after doing core training (Abuwarda, 2014). Indeed, the stronger core muscles not only more economically and harmoniously transfer the strength of athletes to the limbs, but also better maintain the stability of the trunk and hip joints so that gymnasts can show more coherent, coordinated, and stable complex technical movements in the air (Yang & Li, 2010).

### 4.4 Effect of Core Training on Net/Court Sports

According to one study, core training can help volleyball athletes enhance their serving accuracy and velocity of service performance (Yapici, 2019). The overwhelming evidence established that volleyball athletes gain ball speed as a result of their ability to produce speed using their striking hand (Vint & Hinrichs, 2004). Meanwhile, the speed with which the hand strikes is largely determined by the way the movement chain is executed, which includes the trunk, shoulders, hips, elbow, and wrist (Gutiérrez et al., 1994; Rokito et al., 1998). The reason behind these improved skills could be explained through the role of the core in movement chain theory, core muscles function as hubs in the biological motor chain, which create a fulcrum for the strength of the four limbs, and establish a channel for cohesion, transmission, and integration of the upper and lower limbs (Anant & Venugopal, 2021). In addition, strong core muscles establish a stable platform for athletes in fast and changeable sports, increase the stability and control of athletes’ dynamic and static posture, and ensure the success rate of special skills (Allen et al., 2002).

On the other hand, smashing ability is critical to winning points in badminton and is primarily determined by two factors: smashing velocity as well as accuracy (Brahms, 2014; Grice, 1996). A study discovered that core stability exercise can result in considerable improvements in badminton players’ smash velocity and accuracy (Hassan, 2017). This could be explained by the core’s function in the movement chain, as well as the fact that the smash stroke is dependent on a number of factors, including lower muscular strength, leg power, technique, as well as the correct kinetic chain (Chen et al., 2014; Hassan, 2017; Huang et al., 2002; Kimura et al., 2014).

### 4.5 Effect of Core Training on Invasion Sports

Some studies showed that core straining could improve the skills in terms of particular throwing types (Manchado et al., 2017), dribbling, fast passing, shooting (Akçinar & Macit, 2020), long jump shot, fly distance, and long jump shot accuracy significantly (Mahmoud, 2021). Some studies examined the muscles’ skill, coordination, as well as maximum explosive power in the lower and upper body are the essential factors in determining throwing quality (Van Muijen et al., 1991; Manchado et al., 2013). Some studies provided reasons that throwing, shooting, and passing are done by transferring the force created from the foot muscles through the trunk to end with the ball leaving the shooting palm, which requires the movement transfer in which the force is transferred between the body parts. Thus, a strong core could better control and transfer motion and force to the terminal segment, ensuring that those skills are more robust and faster (Kibler et al., 2006; Azizi, 2009).

Two studies provided evidence that core training could improve many basketball skills (Dogan & Savaş, 2021; Şahiner & Koca, 2021). Basketball is a sport that requires athletes to change their body positions constantly. Athletes need to maintain control and balance when using offensive or defensive skills, so they need to stimulate the coordinated movement of multiple muscles through the core area (McGill, 2006). Thus, it is thought that the vital core could assist the movement transfer during the movement can impact the skill of the basketball players (Lee & McGill, 2015). Some studies showed that solid core muscles provide the ability of balance and postural control and could help athletes acquire and practice skills easily (McGill, 2010; Mayda et al., 2016). Meanwhile, a robust core muscular system could allow motor control and prepare for gravity or torque from an opponent and dynamic reactions (Willardson, 2014).

Additionally, several research found that core training had the same beneficial effects on footballers (Bavli & Koç, 2018; Mahmoud, 2018; Ahmed et al., 2021). The body’s crucial core region is regarded as critical for efficient biomechanics. It enables excellent force and motion production, transference, and control to the terminal section during integrated athletic activities. Core muscle activity can be defined as the preprogrammed integration of local, single-joint, and multi-joint muscles for the purpose of providing stability and producing motion. This results in proximal stability for distal mobility, a proximal to distal patterning of force generation, and the formation of interactive moments that move and protect distal joints. Simultaneously, interactive moments maximise force at the distal end while maintaining precision and stability at the distal tip (Kibler et al., 2006). As a result, this clarified why football players might benefit from core training.

### 4.6 Effect of Core Training on Combat Sports

Two studies examined that core training could help Muay Thai and karate athletes improve their skill performance (Kamal, 2015; Lee & McGill, 2017). One study examined that balance is the key to Karate players’ achievement (Fabio, 2004). A play who can perform offensive and defensive skills with balance can be considered a high-level player. The ability to control physiological balance is derived from the vestibular receptors in muscles, tendons, joints, and coordination areas such as visual stimulation (Hibbs et al., 2008). Core training timely controls the spine and pelvis stability, coordinates the players’ changing center of gravity and posture adjustment during consistent movement, and improves core stability. Consequently, balance could be improved (Hu & Li, 2019).

According to another study, Muay Thai players’ objective is to deliver a high-impact force at a high velocity to an intended target. These athletes have long sought the optimal method of enhancing their amazing speed, impact force, as well as other performance indicators. The body could be viewed as an articulated linkage in which proximal stiffness as well as stability are required to allow distal segments to move rapidly (McGill, 2010). When working against external stressors, the core muscles may increase spinal rigidity as well as stability to prevent undesired torso motion (Bergmark, 1989). Isometric core training has been shown to increase torso stiffness and effective mass, allowing players to transmit greater force of impact while minimizing or “stiffening out” the torso eccentric micro-movements. Stiffer core muscles that stabilize the spine tend to be more effective at preventing “energy leakage” (McGill, 2006). It appears as though one could explain why core training increased impact force. However, it remains unknown why isometric core training increased impact force, while dynamic core training increased strike velocity.

## 5 Limitations

Although this review provided evidence to examine the effect of core training on skill performance among athletes, some weaknesses still identified from this review are outlined as follows:

1) Lack of athletes from other sports fields as participants.
2) The existing literature focuses on the comparison between core training and traditional or daily training, and lacks comparison with other new training methods, such as functional training.
3) In the existing literature, the impact of core training on the performance of different sports skills is not comprehensive, and only focuses on some sports skills.
4) The existing literature ignores the particularity of athletes’ positions and weight levels, which may lead to a deviation in the measurement results.

## 6 Conclusion

This review provided evidence that core training could potentially improve some skill performance of football, handball, basketball, swimming, dancing, Karate, Muay Thai, gymnasts, volleyball, badminton, and golf players.

However, based on the above sixteen articles, the existing literature only studied the impact of core training on some sports, and lacks consideration of fast strength sports and endurance sports. Therefore, researchers can continue to explore these gaps to help athletes achieve better skill performance in competitions.

## 7 Practical Application

In almost all sports, athletes cannot rely on a single joint or muscle group to apply their skills. Movement requires the local and global coordination of various muscle groups required by multi-joint coordination. Compared with the traditional training methods, core training is a new strength training method. And the effect on the core muscles can be assessed by medicine ball throwing (in different directions), McGill’s core assessment (maintain a static muscle contraction for an extended period of time in 4 different positions), and the star excursion balance test.

Strong core muscles function as hubs in the biological motor chain, which create a fulcrum for the four limbs’ strength and establish a channel for the cohesion, transmission, and integration of the upper and lower limbs. In other words, core training optimizes the production, control and transfer of force and motion to the terminal segment within integrated athletic events. Meanwhile, core training could increase spinal stiffness as well as stability to avoid unrequired torso motion and “energy leaks” during exertion against incoming loads. This mechanism could help athletes achieve better skill performance and reduce the risk of injury.

Finally, based on the above sixteen studies, this review suggested that core training should be considered integrated into athletes’ daily training and the frequency and duration could not be less than 2 times a week and 4 weeks.

## Data Availability

The original contributions presented in the study are included in the article/Supplementary Material, further inquiries can be directed to the corresponding authors.
